# Supplementary material for: Pyruvate Dehydrogenase Kinase 1 inhibition mediated oxidative phosphorylation enhancement in cartilage promotes osteoarthritis progression
Source: BMC Musculoskelet Disord. 2023 Jul 20;24:597. doi: 10.1186/s12891-023-06585-6 (PMC10357736; doi:10.1186/s12891-023-06585-6)
Supplement: Supplementary file 1 — Additional file 1. [file 12891_2023_6585_MOESM1_ESM.zip › MF.Gsea.1653627316242/GOMF_CARBOXYLIC_ESTER_HYDROLASE_ACTIVITY.html]

Details for gene set GOMF\_CARBOXYLIC\_ESTER\_HYDROLASE\_ACTIVITY[GSEA]

|  || Dataset | 55457\_collapsed\_to\_symbols.cls.cls #OA\_versus\_normal.cls.cls #OA\_versus\_normal\_repos |
| Phenotype | cls.cls#OA\_versus\_normal\_repos |
| Upregulated in class | OA |
| GeneSet | GOMF\_CARBOXYLIC\_ESTER\_HYDROLASE\_ACTIVITY |
| Enrichment Score (ES) | 0.24459669 |
| Normalized Enrichment Score (NES) | 1.1292678 |
| Nominal p-value | 0.19414894 |
| FDR q-value | 0.64049536 |
| FWER p-Value | 1.0 |
Table: GSEA Results Summary

  

Fig 1: Enrichment plot: GOMF\_CARBOXYLIC\_ESTER\_HYDROLASE\_ACTIVITY      
 Profile of the Running ES Score & Positions of GeneSet Members on the Rank Ordered List

  

| SYMBOL | TITLE | RANK IN GENE LIST | RANK METRIC SCORE | RUNNING ES | CORE ENRICHMENT || 1 | GDE1 | glycerophosphodiester phosphodiesterase 1 [Source:HGNC Symbol;Acc:HGNC:29644] | 31 | 1.039 | 0.0336 | Yes |
| 2 | PNPLA4 | patatin like phospholipase domain containing 4 [Source:HGNC Symbol;Acc:HGNC:24887] | 65 | 0.963 | 0.0644 | Yes |
| 3 | RPE65 | retinoid isomerohydrolase RPE65 [Source:HGNC Symbol;Acc:HGNC:10294] | 308 | 0.743 | 0.0713 | Yes |
| 4 | ABHD10 | "abhydrolase domain containing 10, depalmitoylase [Source:HGNC Symbol;Acc:HGNC:25656]" | 437 | 0.690 | 0.0852 | Yes |
| 5 | LIPC | "lipase C, hepatic type [Source:HGNC Symbol;Acc:HGNC:6619]" | 457 | 0.678 | 0.1072 | Yes |
| 6 | ESD | esterase D [Source:HGNC Symbol;Acc:HGNC:3465] | 529 | 0.647 | 0.1241 | Yes |
| 7 | PLA2G12A | phospholipase A2 group XIIA [Source:HGNC Symbol;Acc:HGNC:18554] | 600 | 0.623 | 0.1402 | Yes |
| 8 | ABHD8 | abhydrolase domain containing 8 [Source:HGNC Symbol;Acc:HGNC:23759] | 746 | 0.582 | 0.1491 | Yes |
| 9 | CES1 | carboxylesterase 1 [Source:HGNC Symbol;Acc:HGNC:1863] | 918 | 0.537 | 0.1544 | Yes |
| 10 | ACOT7 | acyl-CoA thioesterase 7 [Source:HGNC Symbol;Acc:HGNC:24157] | 971 | 0.524 | 0.1685 | Yes |
| 11 | PGLS | 6-phosphogluconolactonase [Source:HGNC Symbol;Acc:HGNC:8903] | 976 | 0.523 | 0.1863 | Yes |
| 12 | ALDH2 | aldehyde dehydrogenase 2 family member [Source:HGNC Symbol;Acc:HGNC:404] | 1003 | 0.517 | 0.2022 | Yes |
| 13 | PLAAT4 | phospholipase A and acyltransferase 4 [Source:HGNC Symbol;Acc:HGNC:9869] | 1019 | 0.513 | 0.2188 | Yes |
| 14 | ACOT8 | acyl-CoA thioesterase 8 [Source:HGNC Symbol;Acc:HGNC:15919] | 1088 | 0.500 | 0.2309 | Yes |
| 15 | MRPL58 | mitochondrial ribosomal protein L58 [Source:HGNC Symbol;Acc:HGNC:5359] | 1298 | 0.459 | 0.2304 | Yes |
| 16 | RGN | regucalcin [Source:HGNC Symbol;Acc:HGNC:9989] | 1369 | 0.448 | 0.2405 | Yes |
| 17 | IARS2 | "isoleucyl-tRNA synthetase 2, mitochondrial [Source:HGNC Symbol;Acc:HGNC:29685]" | 1685 | 0.401 | 0.2298 | Yes |
| 18 | PLA2G15 | phospholipase A2 group XV [Source:HGNC Symbol;Acc:HGNC:17163] | 1811 | 0.382 | 0.2333 | Yes |
| 19 | LIPA | "lipase A, lysosomal acid type [Source:HGNC Symbol;Acc:HGNC:6617]" | 1836 | 0.380 | 0.2446 | Yes |
| 20 | PON2 | paraoxonase 2 [Source:HGNC Symbol;Acc:HGNC:9205] | 2292 | 0.328 | 0.2204 | No |
| 21 | BCHE | butyrylcholinesterase [Source:HGNC Symbol;Acc:HGNC:983] | 2455 | 0.308 | 0.2184 | No |
| 22 | LGALS13 | galectin 13 [Source:HGNC Symbol;Acc:HGNC:15449] | 2467 | 0.307 | 0.2282 | No |
| 23 | ACHE | acetylcholinesterase (Cartwright blood group) [Source:HGNC Symbol;Acc:HGNC:108] | 2642 | 0.286 | 0.2245 | No |
| 24 | PLA2G4A | phospholipase A2 group IVA [Source:HGNC Symbol;Acc:HGNC:9035] | 2656 | 0.284 | 0.2334 | No |
| 25 | AADAC | arylacetamide deacetylase [Source:HGNC Symbol;Acc:HGNC:17] | 2740 | 0.276 | 0.2365 | No |
| 26 | CES3 | carboxylesterase 3 [Source:HGNC Symbol;Acc:HGNC:1865] | 2905 | 0.260 | 0.2327 | No |
| 27 | PGAP6 | post-glycosylphosphatidylinositol attachment to proteins 6 [Source:HGNC Symbol;Acc:HGNC:17205] | 2928 | 0.258 | 0.2399 | No |
| 28 | PAFAH1B2 | platelet activating factor acetylhydrolase 1b catalytic subunit 2 [Source:HGNC Symbol;Acc:HGNC:8575] | 3151 | 0.235 | 0.2307 | No |
| 29 | PNLIPRP2 | pancreatic lipase related protein 2 (gene/pseudogene) [Source:HGNC Symbol;Acc:HGNC:9157] | 3268 | 0.224 | 0.2294 | No |
| 30 | PLA2G10 | phospholipase A2 group X [Source:HGNC Symbol;Acc:HGNC:9029] | 3315 | 0.220 | 0.2334 | No |
| 31 | PLA2G3 | phospholipase A2 group III [Source:HGNC Symbol;Acc:HGNC:17934] | 3648 | 0.190 | 0.2141 | No |
| 32 | CES2 | carboxylesterase 2 [Source:HGNC Symbol;Acc:HGNC:1864] | 3689 | 0.186 | 0.2174 | No |
| 33 | AOAH | acyloxyacyl hydrolase [Source:HGNC Symbol;Acc:HGNC:548] | 3978 | 0.158 | 0.2004 | No |
| 34 | PLAAT3 | phospholipase A and acyltransferase 3 [Source:HGNC Symbol;Acc:HGNC:17825] | 4013 | 0.155 | 0.2031 | No |
| 35 | MGLL | monoglyceride lipase [Source:HGNC Symbol;Acc:HGNC:17038] | 4018 | 0.154 | 0.2082 | No |
| 36 | ACOT2 | acyl-CoA thioesterase 2 [Source:HGNC Symbol;Acc:HGNC:18431] | 4046 | 0.152 | 0.2113 | No |
| 37 | PAFAH2 | platelet activating factor acetylhydrolase 2 [Source:HGNC Symbol;Acc:HGNC:8579] | 4446 | 0.118 | 0.1842 | No |
| 38 | ABHD6 | "abhydrolase domain containing 6, acylglycerol lipase [Source:HGNC Symbol;Acc:HGNC:21398]" | 4935 | 0.077 | 0.1488 | No |
| 39 | PON1 | paraoxonase 1 [Source:HGNC Symbol;Acc:HGNC:9204] | 5031 | 0.071 | 0.1438 | No |
| 40 | PLA2G2D | phospholipase A2 group IID [Source:HGNC Symbol;Acc:HGNC:9033] | 5056 | 0.069 | 0.1443 | No |
| 41 | PON3 | paraoxonase 3 [Source:HGNC Symbol;Acc:HGNC:9206] | 5340 | 0.045 | 0.1238 | No |
| 42 | ABHD4 | "abhydrolase domain containing 4, N-acyl phospholipase B [Source:HGNC Symbol;Acc:HGNC:20154]" | 5346 | 0.044 | 0.1249 | No |
| 43 | NDST3 | N-deacetylase and N-sulfotransferase 3 [Source:HGNC Symbol;Acc:HGNC:7682] | 5446 | 0.037 | 0.1185 | No |
| 44 | ACOT9 | acyl-CoA thioesterase 9 [Source:HGNC Symbol;Acc:HGNC:17152] | 5451 | 0.037 | 0.1194 | No |
| 45 | ABHD3 | "abhydrolase domain containing 3, phospholipase [Source:HGNC Symbol;Acc:HGNC:18718]" | 5456 | 0.037 | 0.1204 | No |
| 46 | TARS2 | "threonyl-tRNA synthetase 2, mitochondrial [Source:HGNC Symbol;Acc:HGNC:30740]" | 5576 | 0.026 | 0.1120 | No |
| 47 | PLA2G4C | phospholipase A2 group IVC [Source:HGNC Symbol;Acc:HGNC:9037] | 5677 | 0.017 | 0.1048 | No |
| 48 | AARS1 | alanyl-tRNA synthetase 1 [Source:HGNC Symbol;Acc:HGNC:20] | 5703 | 0.015 | 0.1034 | No |
| 49 | PLA1A | phospholipase A1 member A [Source:HGNC Symbol;Acc:HGNC:17661] | 5987 | -0.003 | 0.0814 | No |
| 50 | PAFAH1B3 | platelet activating factor acetylhydrolase 1b catalytic subunit 3 [Source:HGNC Symbol;Acc:HGNC:8576] | 6060 | -0.009 | 0.0761 | No |
| 51 | BPHL | biphenyl hydrolase like [Source:HGNC Symbol;Acc:HGNC:1094] | 6262 | -0.025 | 0.0612 | No |
| 52 | DAGLA | diacylglycerol lipase alpha [Source:HGNC Symbol;Acc:HGNC:1165] | 6432 | -0.037 | 0.0493 | No |
| 53 | DDHD2 | DDHD domain containing 2 [Source:HGNC Symbol;Acc:HGNC:29106] | 6517 | -0.043 | 0.0443 | No |
| 54 | PLA2G5 | phospholipase A2 group V [Source:HGNC Symbol;Acc:HGNC:9038] | 6772 | -0.061 | 0.0265 | No |
| 55 | PRDX6 | peroxiredoxin 6 [Source:HGNC Symbol;Acc:HGNC:16753] | 6783 | -0.061 | 0.0279 | No |
| 56 | TNFAIP6 | TNF alpha induced protein 6 [Source:HGNC Symbol;Acc:HGNC:11898] | 6799 | -0.063 | 0.0289 | No |
| 57 | PNPLA6 | patatin like phospholipase domain containing 6 [Source:HGNC Symbol;Acc:HGNC:16268] | 6849 | -0.067 | 0.0274 | No |
| 58 | LARS2 | "leucyl-tRNA synthetase 2, mitochondrial [Source:HGNC Symbol;Acc:HGNC:17095]" | 7017 | -0.081 | 0.0172 | No |
| 59 | APMAP | adipocyte plasma membrane associated protein [Source:HGNC Symbol;Acc:HGNC:13238] | 7117 | -0.089 | 0.0125 | No |
| 60 | LIPE | "lipase E, hormone sensitive type [Source:HGNC Symbol;Acc:HGNC:6621]" | 7185 | -0.095 | 0.0106 | No |
| 61 | PPME1 | protein phosphatase methylesterase 1 [Source:HGNC Symbol;Acc:HGNC:30178] | 7483 | -0.117 | -0.0086 | No |
| 62 | PLA2G2E | phospholipase A2 group IIE [Source:HGNC Symbol;Acc:HGNC:13414] | 7486 | -0.117 | -0.0047 | No |
| 63 | CA1 | carbonic anhydrase 1 [Source:HGNC Symbol;Acc:HGNC:1368] | 7585 | -0.126 | -0.0079 | No |
| 64 | CA2 | carbonic anhydrase 2 [Source:HGNC Symbol;Acc:HGNC:1373] | 7708 | -0.135 | -0.0128 | No |
| 65 | ACOT11 | acyl-CoA thioesterase 11 [Source:HGNC Symbol;Acc:HGNC:18156] | 7746 | -0.137 | -0.0109 | No |
| 66 | PLA2G1B | phospholipase A2 group IB [Source:HGNC Symbol;Acc:HGNC:9030] | 8232 | -0.173 | -0.0428 | No |
| 67 | H6PD | hexose-6-phosphate dehydrogenase/glucose 1-dehydrogenase [Source:HGNC Symbol;Acc:HGNC:4795] | 8303 | -0.178 | -0.0421 | No |
| 68 | LPL | lipoprotein lipase [Source:HGNC Symbol;Acc:HGNC:6677] | 8374 | -0.184 | -0.0412 | No |
| 69 | CEL | carboxyl ester lipase [Source:HGNC Symbol;Acc:HGNC:1848] | 8375 | -0.184 | -0.0348 | No |
| 70 | ENPP2 | ectonucleotide pyrophosphatase/phosphodiesterase 2 [Source:HGNC Symbol;Acc:HGNC:3357] | 8442 | -0.189 | -0.0334 | No |
| 71 | PNPLA3 | patatin like phospholipase domain containing 3 [Source:HGNC Symbol;Acc:HGNC:18590] | 8455 | -0.190 | -0.0277 | No |
| 72 | PNLIPRP1 | pancreatic lipase related protein 1 [Source:HGNC Symbol;Acc:HGNC:9156] | 8798 | -0.216 | -0.0470 | No |
| 73 | LYPLA1 | lysophospholipase 1 [Source:HGNC Symbol;Acc:HGNC:6737] | 9023 | -0.234 | -0.0563 | No |
| 74 | LIPF | "lipase F, gastric type [Source:HGNC Symbol;Acc:HGNC:6622]" | 9074 | -0.238 | -0.0520 | No |
| 75 | ABHD2 | "abhydrolase domain containing 2, acylglycerol lipase [Source:HGNC Symbol;Acc:HGNC:18717]" | 9119 | -0.241 | -0.0471 | No |
| 76 | PLAAT1 | phospholipase A and acyltransferase 1 [Source:HGNC Symbol;Acc:HGNC:14922] | 9264 | -0.253 | -0.0496 | No |
| 77 | PGAP1 | post-GPI attachment to proteins inositol deacylase 1 [Source:HGNC Symbol;Acc:HGNC:25712] | 9358 | -0.260 | -0.0478 | No |
| 78 | PLA2G7 | phospholipase A2 group VII [Source:HGNC Symbol;Acc:HGNC:9040] | 9467 | -0.271 | -0.0469 | No |
| 79 | PTRH2 | peptidyl-tRNA hydrolase 2 [Source:HGNC Symbol;Acc:HGNC:24265] | 9707 | -0.293 | -0.0554 | No |
| 80 | FAAH | fatty acid amide hydrolase [Source:HGNC Symbol;Acc:HGNC:3553] | 9787 | -0.300 | -0.0511 | No |
| 81 | PNPLA2 | patatin like phospholipase domain containing 2 [Source:HGNC Symbol;Acc:HGNC:30802] | 9870 | -0.309 | -0.0468 | No |
| 82 | PLA2G2A | phospholipase A2 group IIA [Source:HGNC Symbol;Acc:HGNC:9031] | 10152 | -0.336 | -0.0571 | No |
| 83 | PLAAT2 | phospholipase A and acyltransferase 2 [Source:HGNC Symbol;Acc:HGNC:17824] | 10153 | -0.337 | -0.0454 | No |
| 84 | IARS1 | isoleucyl-tRNA synthetase 1 [Source:HGNC Symbol;Acc:HGNC:5330] | 10154 | -0.337 | -0.0338 | No |
| 85 | LIPG | "lipase G, endothelial type [Source:HGNC Symbol;Acc:HGNC:6623]" | 10181 | -0.339 | -0.0240 | No |
| 86 | AARSD1 | alanyl-tRNA synthetase domain containing 1 [Source:HGNC Symbol;Acc:HGNC:28417] | 10222 | -0.343 | -0.0153 | No |
| 87 | BAAT | bile acid-CoA:amino acid N-acyltransferase [Source:HGNC Symbol;Acc:HGNC:932] | 10523 | -0.376 | -0.0257 | No |
| 88 | ABHD5 | "abhydrolase domain containing 5, lysophosphatidic acid acyltransferase [Source:HGNC Symbol;Acc:HGNC:21396]" | 10563 | -0.380 | -0.0155 | No |
| 89 | PLA2G2F | phospholipase A2 group IIF [Source:HGNC Symbol;Acc:HGNC:30040] | 10628 | -0.386 | -0.0072 | No |
| 90 | LYPLA2 | lysophospholipase 2 [Source:HGNC Symbol;Acc:HGNC:6738] | 10860 | -0.411 | -0.0110 | No |
| 91 | CLC | Charcot-Leyden crystal galectin [Source:HGNC Symbol;Acc:HGNC:2014] | 11012 | -0.431 | -0.0078 | No |
| 92 | VARS1 | valyl-tRNA synthetase 1 [Source:HGNC Symbol;Acc:HGNC:12651] | 11035 | -0.435 | 0.0056 | No |
| 93 | PLA2G6 | phospholipase A2 group VI [Source:HGNC Symbol;Acc:HGNC:9039] | 11128 | -0.446 | 0.0139 | No |
| 94 | NDST1 | N-deacetylase and N-sulfotransferase 1 [Source:HGNC Symbol;Acc:HGNC:7680] | 11241 | -0.462 | 0.0211 | No |
| 95 | PNLIP | pancreatic lipase [Source:HGNC Symbol;Acc:HGNC:9155] | 11329 | -0.475 | 0.0308 | No |
| 96 | LCAT | lecithin-cholesterol acyltransferase [Source:HGNC Symbol;Acc:HGNC:6522] | 11514 | -0.503 | 0.0339 | No |
| 97 | GDPD3 | glycerophosphodiester phosphodiesterase domain containing 3 [Source:HGNC Symbol;Acc:HGNC:28638] | 12299 | -0.676 | -0.0039 | No |
| 98 | LARS1 | leucyl-tRNA synthetase 1 [Source:HGNC Symbol;Acc:HGNC:6512] | 12415 | -0.712 | 0.0118 | No |
| 99 | CHKA | choline kinase alpha [Source:HGNC Symbol;Acc:HGNC:1937] | 12511 | -0.751 | 0.0304 | No |
Table: GSEA details [plain text format]

  

Fig 2: GOMF\_CARBOXYLIC\_ESTER\_HYDROLASE\_ACTIVITY      
 Blue-Pink O' Gram in the Space of the Analyzed GeneSet

  

Fig 3: GOMF\_CARBOXYLIC\_ESTER\_HYDROLASE\_ACTIVITY: Random ES distribution      
 Gene set null distribution of ES for **GOMF\_CARBOXYLIC\_ESTER\_HYDROLASE\_ACTIVITY**
